# *BnaA03.ANS* Identified by Metabolomics and RNA-seq Partly Played Irreplaceable Role in Pigmentation of Red Rapeseed (*Brassica napus*) Petal

**DOI:** 10.3389/fpls.2022.940765

**Published:** 2022-07-14

**Authors:** Pengfei Hao, Han Liu, Baogang Lin, Yun Ren, Lan Huang, Lixi Jiang, Shuijin Hua

**Affiliations:** ^1^Institute of Crops and Nuclear Technology Utilization, Zhejiang Academy of Agricultural Sciences, Hangzhou, China; ^2^Yongding Agriculture and Rural Bureau of Longyan, Longyan, China; ^3^Huzhou Agricultural Science and Technology Development Center/Huzhou Academy of Agricultural Sciences, Huzhou, China; ^4^College of Agriculture and Biotechnology, Zhejiang University, Hangzhou, China

**Keywords:** carotenoids, metabolomics, overexpression, RNA interference, RNA-seq

## Abstract

Colorful flowers of rapeseed (*Brassica napus* L.) have been a hotspot for researchers, but the underlying mechanisms of pigment formation still need to be clarified. In this study, two stages of unopened rapeseed petals with red, white, and yellow colors were selected to identify the metabolites and genes involved in red pigment formation. Metabolomic analysis showed that flavonoids enriched the most co-differentially accumulated metabolites among all categories, and showed higher accumulation in red petal rapeseed than in white and yellow petal ones. RNA-seq analysis showed that among co-differentially expressed genes involved in red pigment formation, genes involved in anthocyanin (belonging to flavonoids) biosynthesis pathway were largely regulated by *ANS, DFR*, and *UF3GT*. The expression of those genes was higher in red petals of rapeseed than in white and yellow petals ones as well. Results of RNA interference of *BnaA03.ANS* in red rapeseed altered petal colors from raspberry red to beige red and zinc yellow under different interference levels, with the contents of pelargonidin, cyanidin, lutein, neoxanthin, β-carotene, and lycopene significantly decreased. However, overexpression of *BnaA03.ANS* in yellow rapeseed petals did not change the color of yellow petals. This study confirmed the important function of flavonoids, especially anthocyanins on red pigment formation, and for the first time, identified the irreplaceable role of *BnaA03.ANS* on red-flowered rapeseed.

## Introduction

*Brassica napus* L. (AACC, 2*n* = 38), an allopolyploid species derived from the hybridization of *Brassica rapa* (AA, 2*n* = 20) and *Brassica oleracea* (CC, 2*n* = 18), is an important cash crop not only showing edible and industrial oil purpose but also exhibiting great ornamental value (Chalhoub et al., 2014; Fu et al., 2016). For example, Hubei Province, China, owns over 25.68 million hectares of rapeseed, the tourism of which caused by rapeseed “flower sea” has contributed to more than half of the county's GDP in 2011 (Fu et al., 2016).

Flower color is critical for ornamental and landscaping utilization by providing aesthetically pleasing scenes. The traditional flower color of rapeseed is yellow and some mutants with light to dark yellow, and with the exploitation of the ornamental values, various petal colors were bred, such as white, orange, pink, red, and purple (Yin et al., 2019). However, the mechanisms for regulating different petal colorations are still unclear.

Carotenoids, flavonoids (namely, anthocyanins, flavones, and flavonols), and betalain biosynthesis are reported as the three most important pathways which produce secondary metabolites that contribute to natural petal color display. For instance, carotenoid and betalain pathways contributed to yellow and red, while orange, yellow, red, and blue were attributed to the anthocyanin pathway (Tanaka et al., 2008). More than that, petal tissue structure, epidermal cell shapes, and pH were also reported to influence the formation of petal colors (Vignolini et al., 2015; Zhao and Tao, 2015). Anthocyanins are the main group of flavonoids, and play a crucial role in plant color development, ranging from pink to blue and purple. Over 100 anthocyanins have been identified, primarily originating from six common types, namely, pelargonidin, cyanidin, delphinidin, peonidin, petunidin, and malvidin (Veitch and Grayer, 2008). Previous studies showed that the color differences are highly correlated to different anthocyanin contents and components. By measuring the anthocyanin content of a series of butterfly pea petals, Kazuma et al. (2003) found that the content of anthocyanins was significantly higher in blue petals than in other colors while no anthocyanins were identified in white color petals. As for cineraria, delphinidin and cyanidin mainly contributed to blue and red flower colors, pink flowers were mainly determined by cyanidin and pelargonidin, while delphinidin and cyanidin were the core anthocyanins in purple flowers (Sun et al., 2009). Moreover, Zhang et al. (2011) found that the main pigment deposited in Lagenaria red petals was cyanidins, and pelargonidins primarily leaned toward scarlet.

Based on the irreplaceable role, anthocyanin biosynthesis has risen to be a hotspot of research for plant secondary metabolism and the key genes that participated in its biosynthetic pathway in plants have been clarified (Cheynier et al., 2013). *CHALCONE SYNTHASE* (*CHS*) is the key gene encoding the first enzyme in the anthocyanin biosynthesis pathway, hence influencing the coloration of flower petals. Ectopic expression of *CHS1* gene from Freesia hybrid in petunia altered flower color from white to pink (Sun et al., 2015), and transgenic tobacco plants with *CHS* gene from Malus crabapple showed higher anthocyanin accumulation and a deep red color than the wild-type (Tai et al., 2014). *CHALCONE ISOMERASE* (*CHI*) encodes the second enzyme in anthocyanin biosynthesis and catalyzes the formation of chalcone. The decrease of *CHI* expression level in the petal of asters, tobacco, and carnations was found to lead to a greater accumulation of chalcone and turn the color into yellow (Nishihara et al., 2005). *DIHYDROFLAVONOL 4-REDUCTASE* (*DFR*) is another key gene encoding an enzyme that transfers three types of dihydroflavonols to their corresponding colorless anthocyanins with NADPH. Zhao et al. (2012) found that in different herbaceous peony organs, the highest expression level of *DFR* was observed in petals which accumulated the most anthocyanins, and similar results were also reported in Asian lily and gentian, suggesting the important role of *DFR* in flower color formation (Nakatsuka et al., 2003, 2005). *ANTHOCYANIDIN SYNTHASE* (*ANS*) is a key gene in the late stage of anthocyanin biosynthesis, which catalyzes the leucoanthocyanin to colored anthocyanidin (Heller et al., 1985). It was identified that *ANS* is a small gene family and these genes have been successfully cloned from several ornamental plants, such as *Forsythia supensa*, herbaceous peony, and gerbera (Rosati et al., 1999; Wellmann et al., 2006; Zhao et al., 2012). Rosati et al. (1999) found that null expression of *ANS* in *Forsythia suspensa* resulted in little anthocyanins accumulation in petals, and the absence of expression of *ANS* was found to be the underlying reason for the color change in lisianthus flowers (Shimizu et al., 2011). To date, the cloning and functional verification of genes that control petal color have paved for gene engineering of plants to obtain colorful exhibitions. For example, in *Brassica napus*, Liu et al. (2020) reported that silencing of *BnaA09.Zep* and *BnaC09.Zep* through Crispr/Cas9 confers orange color in *B. napus* petals. By ectopic overexpression of the *OvPAP2* (*Orychophragmus violaceus*) gene, Fu et al. (2018) successfully produced red anthers and petals in yellow oilseed rape. Zhang et al. (2015) reported that disruption of *BnaC03.CCD4* gene enhanced the accumulation of carotenoids leading to the transfer of petal color from white to yellow.

However, how the anthocyanin pathway regulates rapeseed petal color formation and the detailed molecular mechanisms remain unclear, and few studies have reported color-related genes or elaborated on the molecular regulation mechanism underlying anthocyanin-based variation in oilseed rape petal colors (Sagawa et al., 2016; Nikolov, 2019). The function of *ANS*, the key genes in anthocyanin biosynthesis, on flower coloration in *Brassica napus* is not reported in comparison with others. Here, we performed a metabolomics and RNA-seq study on two different stages of unopened petals of red, pure white, and yellow petal rapeseed lines, aiming at elaborating the pigment formation and development mechanisms in *B. napus*. *BnaA03.ANS* was identified as a high expression co-differentially expressed gene (co-DEG) according to RNA-seq and qRT-PCR. Disruption of *BnaA03.ANS* converted rapeseed petal color from raspberry red to beige red or zinc yellow, while overexpression of *BnaA03.ANS* made no change in petal color, but anthocyanins and carotenoids contents showed large differences. This study first comprehensively elaborated on the pigment formation and development mechanisms from genes to metabolites which contributed a lot to the foundation for colorful rapeseed breeding and the function of *BnaA03.ANS* on red color formation was first identified in *B. napus*.

## Materials and Methods

### Plant Materials and Sampling

Three *B. napus* lines with contrasting petal colors were used, which were Zhehuhong (red, abbreviated as V1), Zhehubai (white, abbreviated as V2), and Zheyou 50 (yellow, abbreviated as V3). The transgenic plants with overexpression and RNAi were obtained from Zheyou 50 and Zhehuhong, respectively. The rapeseed plants including the transgenic lines were planted in a glass solar greenhouse with three replications in a completely randomized block design at the Zhejiang Academy of Agricultural Science, China.

At the flowering stage, full plump unopened buds from the main inflorescence with <5 flowers were selected, then all the petals in each bud were separated out, and the developmental stages of unopened petals were defined as follows: stage 1, petals with 5 mm length and colors close to pale; stage 2, petals with 6–7 mm length; stage 3, petals with 8–9 mm length; and stage 4, petals with 10 mm length and were strongly pigmented. The petals in stage 1 and stage 4 of V1, V2, and V3 were utilized for RNA-seq and metabolomic analysis with 3 biological replicates. Petals at all stages were utilized for stereoscopic imaging, and petals at stage 4 were used for carotenoids and anthocyanins quantification. Petal colors were compared according to the RAL method (Long et al., 2011).

### Metabolomic Analysis

Petals at stage 1 and stage 4 of V1, V2, and V3 were prepared, named V1-1, V1-4, V2-1, V2-4, V3-1, and V3-4, respectively, and 3 biological replicates were set. About 50 mg sample was weighed out for supernatant preparation. Agilent 1290 Infinity II UHPLC system coupled to an Agilent 6545 UHD and Accurate-Mass Q-TOF/MS was used for liquid chromatography-mass spectrometry (LC-MS) analysis. The chromatographic column used was Waters XSelect HSS T3 (2.5 μm, 100 mm × 2.1 mm). Raw data were converted to common (mz.data) using Agilent MassHunter Qualitative Analysis B.08.00 software (Agilent Technologies, USA). Then all data went through internal standard normalization and weight normalization. Visualization matrices containing sample name, m/z-RT pair, and peak area were obtained. After editing, the data matrices were imported into SIMCA-P 14.1 (Umetrics, Umea, Sweden), mean-centered and scaled to Pareto variance. Then, a multivariate analysis was conducted. |log_2_FoldChange|>1 and *p* ≤ 0.05 were determined as differentially accumulated metabolites (DAMs). DAMs Venn diagram, principal component analysis (PCA), Gene Ontology (GO), and KEGG enrichment (Kyoto Encyclopedia of Genes and Genomes, KEGG) were also conducted for further analysis.

### RNA-seq Analysis

The preparation of samples was the same as metabolomic analysis. Total RNA was extracted using a polysaccharide and polyphenol total RNA isolation kit (Bioteke, Beijing, China). HISAT2 was used to align all clean reads against the reference genome *Brassicanapus*.Annotation_v4.1. FPKM values were used to calculate the expression level of genes. |log_2_FoldChange >1 and *p* ≤ 0.05 were determined as differentially expressed genes (DEGs). Venn diagram of DEGs, PCA, GO, and KEGG was also conducted for further analysis.

### Quantification of Carotenoids and Anthocyanins Contents

Contents of carotenoids and anthocyanins in 9 rapeseed petals (V1-4, V2-4, V3-4, and each containing 3 biological replicates) were determined through HPLC methods. Carotenoids were performed according to Cao et al. (2012) and the contents of each carotenoid were determined as previously described (Morris et al., 2004).

The components of anthocyanins were determined according to Sun et al. (2014) with several modifications. Approximately 100 mg petal samples were weighed out and 800 μl methanol was added and vortex for 1 min, then shock under 4°C for 30 min. A total of 600 μl supernatant was prepared and concentrated to dry, then 200 μl methanol was added to redissolve the samples. The supernatants were used for LC-MS/MS analysis after centrifuging under 12,000 rpm 4°C for 10 min. The MS parameters were: ESI ion source, 35 arb curtain gas, 7 arb collision gas, 4,500 V ion spray voltage, 450°C temperature, 55 arb ion source gas1, and 55 arb ion source gas2. Multiple reaction monitoring was used for parameter acquisition and MultiQuant software was used for data calculation.

### Plasmid Construction and Transformation

Petal-specific expression promoter *XY355* and the open reading frame of *BnaA03.ANS* was amplified and cloned into the *Pme1*-*Pac1* and *Pac1*-*Asc1* sites of pMDC83, respectively, to construct overexpression plasmid *pXY355::BnaA03.ANS*. The plasmid was introduced into the *Agrobacterium tumefaciens* strain GV3101 and transformed into yellow-flowered rapeseed V3.

The target gene fragments were amplified using *BnaA03.ANS* RNAi primers (Supplementary Table 1), and then ligated into the pNC-Cam1304-RNAi vector using the Nimble Cloning kit (Chinese Academy of Tropical Agricultural Sciences) to construct an *ANS* interference vector. The constructed vector was transformed into *Agrobacterium tumefaciens* GV3101, and the genetic transformation of rapeseed hypocotyl mediated by *Agrobacterium* was used to transform it into red rapeseed V1. Both Nimble Cloning kit and pNC-Cam1304-RNAi vector were donated by Mr. Yan Pu (Chinese Academy of Tropical Agricultural Sciences). The genetic transformation methods were referred to by Zhou et al. (2012).

### qRT-PCR Analysis

Total RNA was extracted from petal samples using a polysaccharide and polyphenol total RNA isolation kit (Bioteke, Beijing, China) and qRT-PCR was performed according to Xu et al. (2017). All primers used in the study were listed in Supplementary Table 1.

### Statistics

Data analysis was performed using IBM SPSS v.22.0 statistical software. Duncan's multiple range test was used to evaluate significant treatment effects at the significance level of *p* ≤ 0.05. MultiQuant software was used to integrate the curves and calculate the contents of anthocyanins and carotenoids according to each standard curve.

## Results

### Phenotype Characterization of all *B. napus* Materials

**Figure 1:**
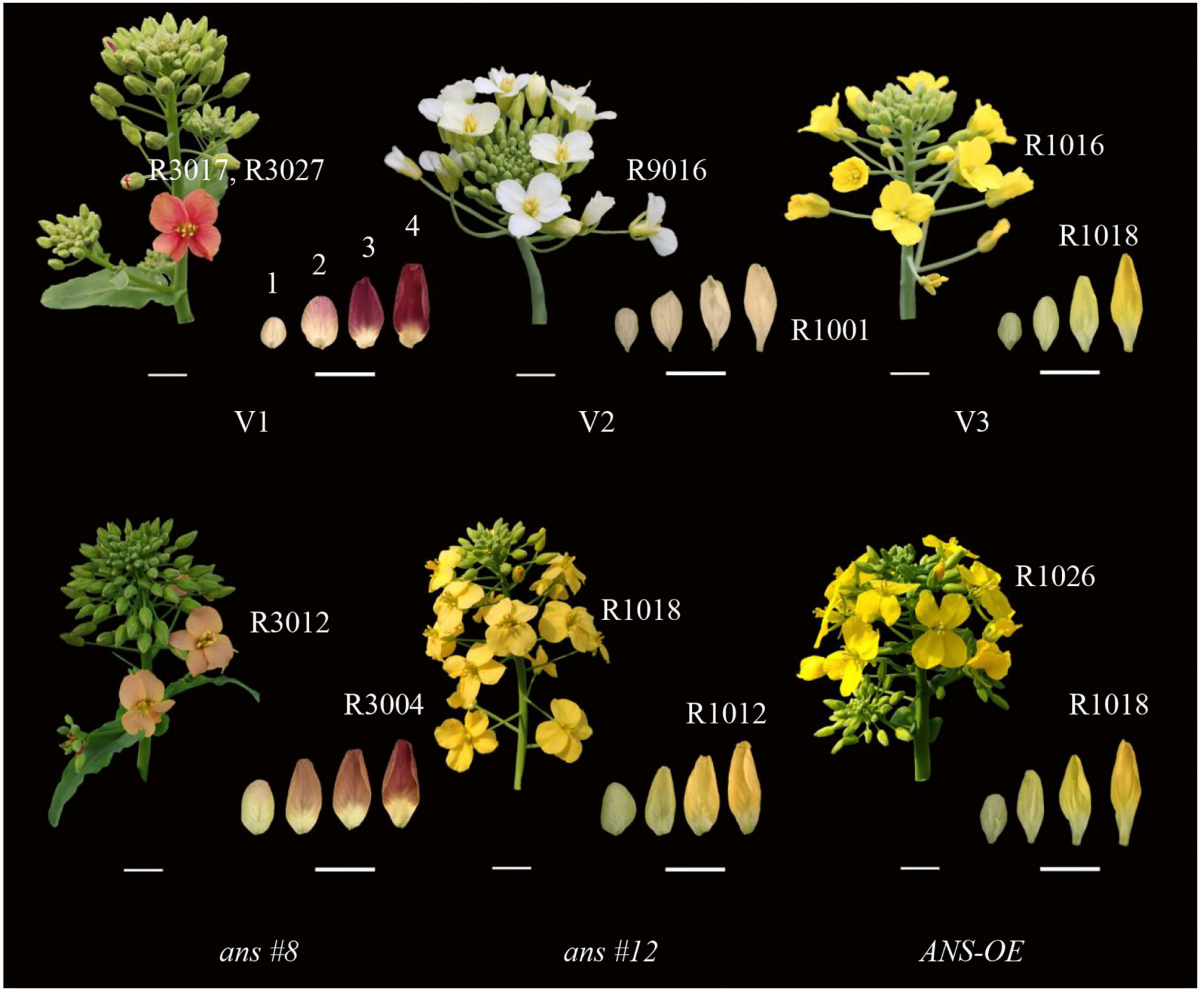
Inflorescence individuals and unopened petals phenotypes of *Brassica napus* with red (Zhehuhong, V1), white (Zhehubai, V2), yellow (Zheyou 50, V3) color, and the transgenic lines (*ans#8* and *ans#12* indicated RNAi-1 and RNAi-2 plants; *ANS-OE indicated* overexpression plants). Numbers on the top of unopened petals in V1 represented 4 development stages. R numbers represent colors alignment to RAL methods. Bars at the left bottom represent 1 cm and bars at the right bottom represent 0.5 cm.

In this experiment, the phenotype of V1, V2, V3, RNAi, and the overexpression lines were recorded (Figure 1). The opened petal color of V1, V2, and V3 matched RAL3017 (rose), RAL9016 (traffic white), and RAL1016 (sulfur yellow), respectively. Different from the edge of the V1 petal, the color of the center matched RAL3027 (raspberry red). While the unopened petals were deeper than those opened petals to some extent, the colors of petals at stage 4 matched RAL4004 (claret violet), RAL1001 (beige), and RAL1018 (zinc yellow), respectively, in V1, V2, and V3.

Petal's color became shallow to some degree in *BnaA03.ANS*RNAi lines, with the color matched to RAL3012 (beige red, count 62.5% of all RNAi lines) and RAL1018 (zinc yellow, count 37.5% of all RNAi lines), while the color of unopened petals at T4 stage matched RAL3004 (purple red) and RAL1012 (lemon yellow), respectively. Overexpression of *BnaA03.ANS* did not alter the unopened petal color with the opened petal color match to RAL1026 (luminous yellow).

### Identification and Functional Analysis of Differentially Accumulated Metabolites in V1, V2, and V3 at Stage 1 and Stage 4

**Figure 2:**
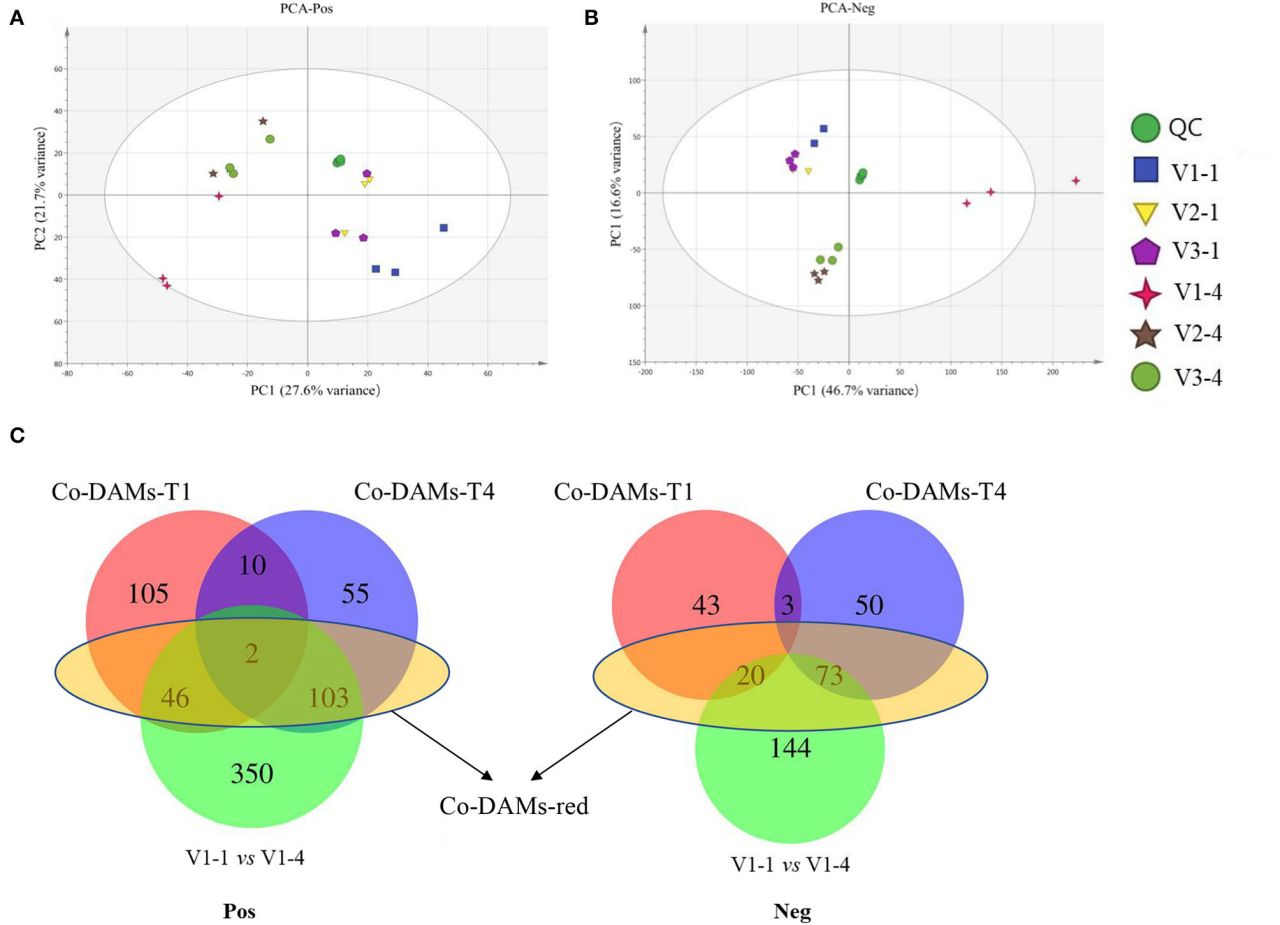
PCA analysis and Venn diagrams of metabolomic data. **(A)** PCA analysis of metabolomic data under positive mode. **(B)** PCA analysis of metabolomic data under negative mode. **(C)** Venn diagram analysis of differentially accumulated metabolites. V1-1, V2-1, and V3-4 represent the unopened petals of V1, V2, and V3 at stage 1. V1-4, V2-4, and V3-4 represent the unopened petals at stage 4. Co-DAMs-T1 represents the common differentially accumulated metabolites between V1 vs. V2 and V1 vs. V3 at stage 1. Co-DAMs-T4 represents the common differentially accumulated metabolites between V1 vs. V2 and V1 vs. V3 at stage 4. Co-DAMs-red represents the metabolites that were considered key metabolites functioning in red color. Pos represents the positive mode and Neg represents the negative mode.

**Figure 3:**
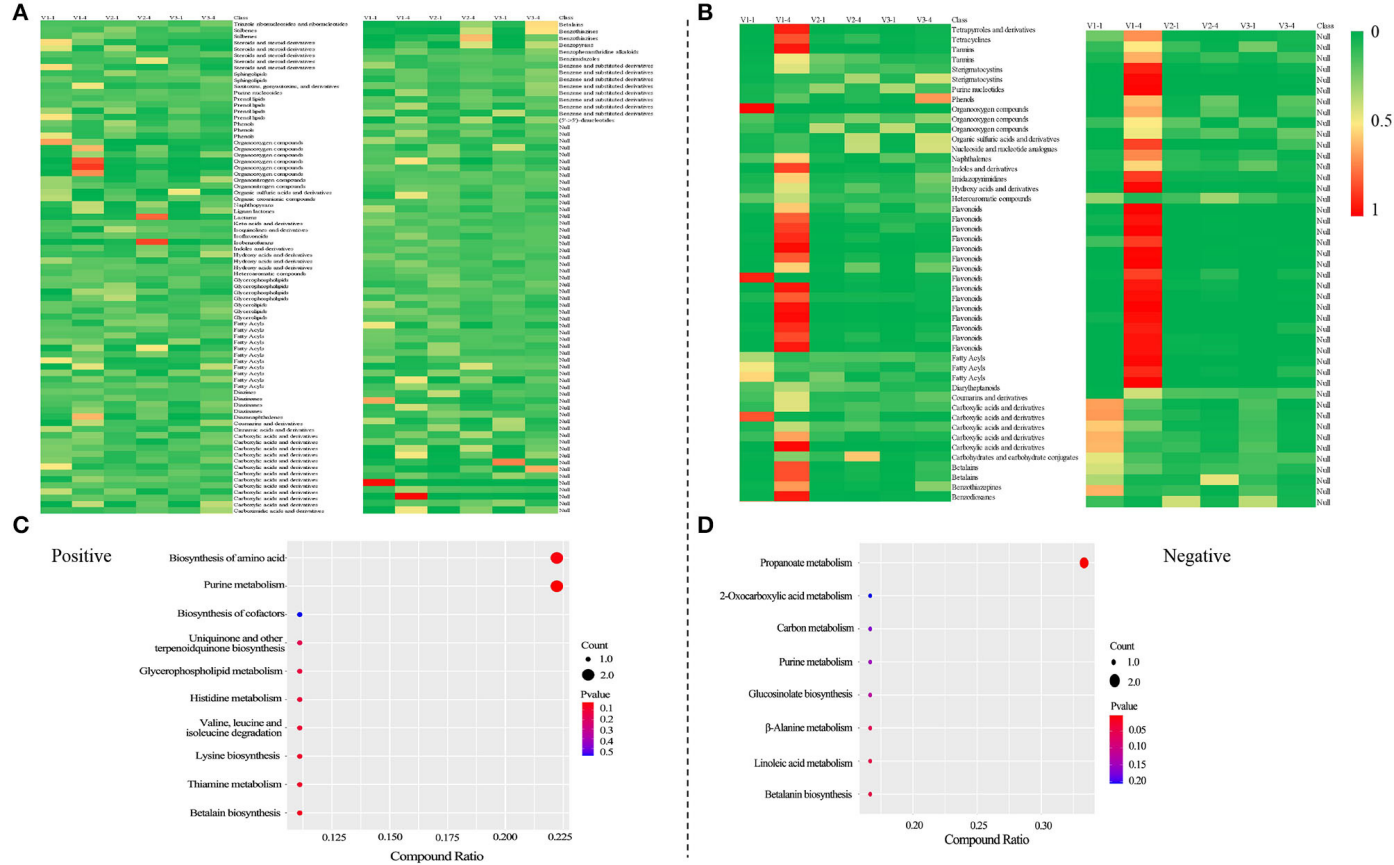
Heatmaps of the accumulation level of differentially accumulated metabolites after normalization and KEGG analysis of Co-DAMs-red. Heatmaps were drawn after normalization, that is take the sum of each metabolite accumulation value in all 6 samples as 1, and calculate the ratio in the sum. **(A,C)** were at positive mode; **(B,D)** were at negative mode.

The result of PCA showed that V1-1 and V1-4 separated significantly from other groups (Figures 2A,B). Under positive mode, a total of 163 and 170 co-DAMs were identified between V1 vs. V2 and V1 vs. V3 at stage 1 and stage 4 (Co-DAMs-T1 and Co-DAMs-T4), respectively. Among them, 46+2+103 metabolites were also co-differentially accumulated in stage 1 and stage 4 of V1, which were determined as Co-DAMs-red metabolites functioning on the red color formation (Figure 2C). The accumulation level of each sample group was shown as a heatmap after normalization processing (Figure 3A), and the top 3 classes were carboxylic acids and derivatives (12 DAMs), fatty acyls (11 DAMs), and benzene and substituted derivatives (8 DAMs). Furthermore, KEGG analysis was performed aiming at these 46+2+103 DAMs, and 10 KEGG pathways were enriched significantly (Figure 3C).

Under negative mode, a total of 66 and 126 co-DAMs were identified between V1 vs. V2 and V1 vs. V3 at stage 1 and stage 4, respectively. Among them, 20+73 metabolites were also co-differentially accumulated at stage 1 and stage 4 of V1 (Figure 2C). The accumulation level of each sample group was shown as a heatmap after normalization processing (Figure 3B), with flavonoids (15 DAMs) being the most class. Furthermore, KEGG analysis was performed aiming at these 20+73 DAMs, and 8 KEGG pathways were enriched significantly (Figure 3D).

### Identification and Functional Analysis of Differentially Expressed Genes in V1, V2, and V3 at Stage 1 and Stage 4

**Figure 4:**
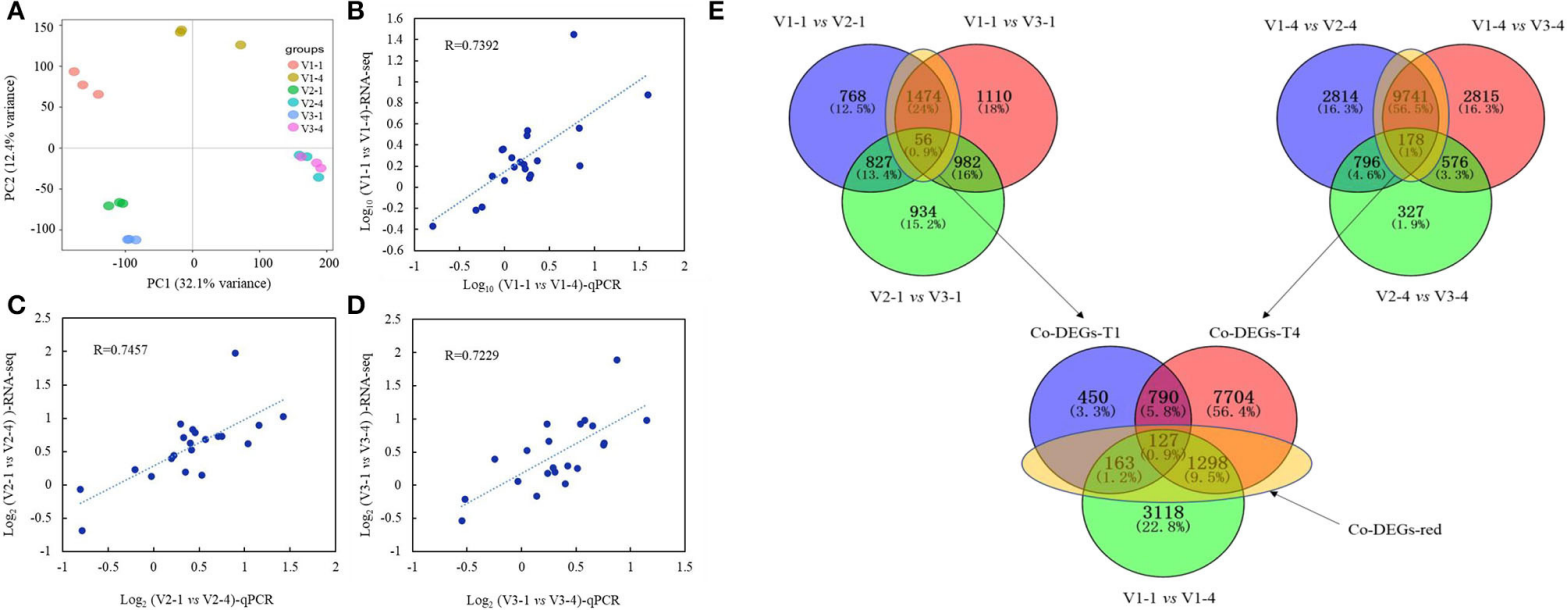
PCA analysis, qRT-PCR validation, and Venn diagrams of RNA-seq data. **(A)** PCA analysis of RNA-seq data; **(B–D)** represent the validation and correlation analysis of RNA-seq and qRT-PCR results of 21 selected genes. **(B)** V1-1 vs. V1-4; C, V2-1 vs. V2-4; D, V3-1 vs. V3-4. **(E)** Venn diagram of common differentially expressed genes (co-DEGs) between V1, V2, and V3 at stage 1 and stage 4. Co-DEGs-T1 represents the genes that are co-differentially expressed in V1 vs. V2 and V1 vs. V3 at stage 1. Co-DEGs-T4 represents the genes that are co-differentially expressed in V1 vs. V2 and V1 vs. V3 at stage 4. Co-DEGs-red represents the genes that were considered key genes that are functioning in red color formation.

For RNA-seq analysis, raw reads, clean reads, total genes, and sequenced genes of each sample were shown in Supplementary Table 2. PCA results showed that these 6 group samples had a distinct separation except for V2 and V3 at stage 4 (Figure 4A). A total of 21 genes were selected randomly to validate the reliability of RNA-seq data by qRT-PCR analysis. Results showed that the relative coefficients of the Log_10_Value of the 21 genes between RNA-seq and qRT-PCR results at group V1-1 vs. V1-4, V2-1 vs. V2-4, and V3-1 vs. V3-4 were 0.7392, 0.7457, and 0.7229, which showed the high reliability of the RNA-seq data (Figures 4B–D).

The result of Venn diagrams showed that at stage 1, 1,530 co-DEGs (1,474+56, named Co-DEGs-T1) were identified between V1 vs. V2 and V1 vs. V3, and a total of 9,919 co-DEGs (9,471+178, named Co-DEGs-T4) were identified between V1 vs. V2 and V1 vs. V3 at stage 4. Among them, 163+127+1,298 co-DEGs were also differentially expressed between Co-DEGs-T1 and Co-DEGs-T4 with V1-1 vs. V1-4. These co-DEGs (163+127+1,298, named DEGs-red) were considered to function on the difference between red with white, red with yellow and light red with deep red petal color (Figure 4E).

### Response of DEGs-Red Participating in Anthocyanin Biosynthesis Pathway, MYB and bHLH Transcription Factors

**Figure 5:**
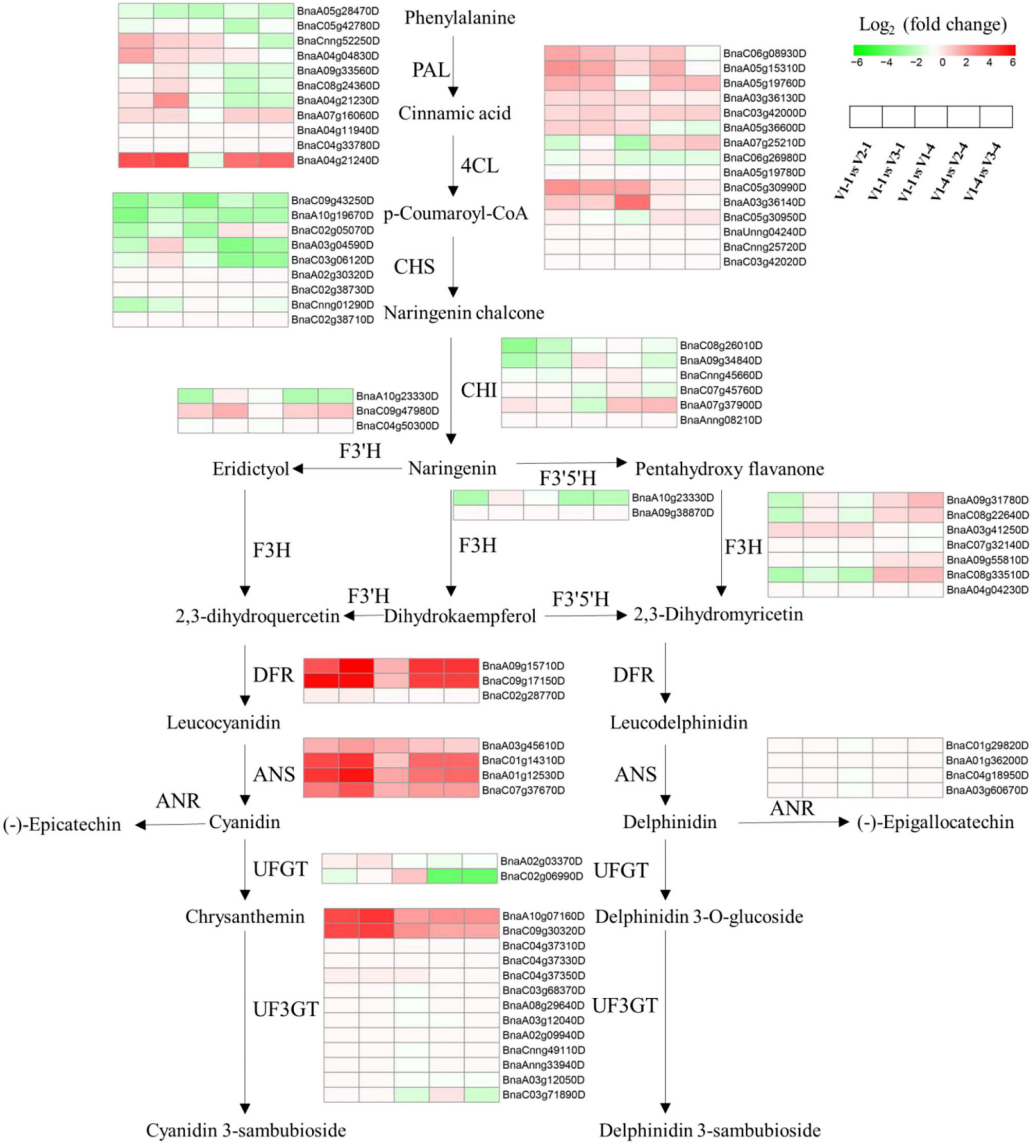
Overview of the transcriptional regulation of anthocyanin biosynthetic genes. Heatmaps represent the log_2_-fold change value of V1-1 vs. V2-1, V1-1 vs. V3-1, V1-1 vs. V1-4, V1-3 vs. V2-4, and V1-4 vs. V3-4, respectively. PAL, phenylalanine ammonia lyase; 4CL, 4-coumarate co ligase; CHS, chalcone synthase; CHI, chalcone isomerase; F3'H, flavonoid 3'-hydroxylase; F3H, flavanone 3-hydroxylase; F3'5'H, flavonoid-3'5'-hydroxylase; DFR, dihydroflavonol reductase; ANS, anthocyanidin synthase; ANR, anthocyanidin reductase; UFGT, uridine diphosphate glucose-flavonoid glucosyltransferase; UF3GT, UDP: flavonoid 3-O-glucosyltransferase.

Aiming at DEGs-red, a total KEGG analysis was conducted and the results were shown in Supplementary Figure 1. Anthocyanin biosynthesis pathway was drawn and the heatmap of relative gene expression level was exhibited. Among them, *ANS* was significantly upregulated in five comparison groups (Figure 5). In addition, MYB, WD40, and bHLH transcription factor family are reported to be the three main transcription factors that affected petal color formation, and the heatmap of MYB and bHLH were shown in Supplementary Figure 2. What's more, qRT-PCR analysis was performed to further verify the expression level of genes relative to petal color (Supplementary Figure 3). Among them, *ANS2, ANS4, PAP2, DFR1, DFR2*, and *UF3GT* genes had significantly higher expression amounts in red petals than in white and yellow at both stage 1 and stage 4. The expression amount of *CHS, CHI, F3H, F3'H*, and *MYB111* was significantly higher in V2 at stage 1 and was higher in V2 at stage 4 for *PAP2* and *MBY61* while it was higher in V3 at stage 1 for *MYB5* and *MYC1*.

### Influence of RNAi and Overexpression of *BnaA03.ANS* on Petal Colors and Pigment Contents

**Figure 6:**
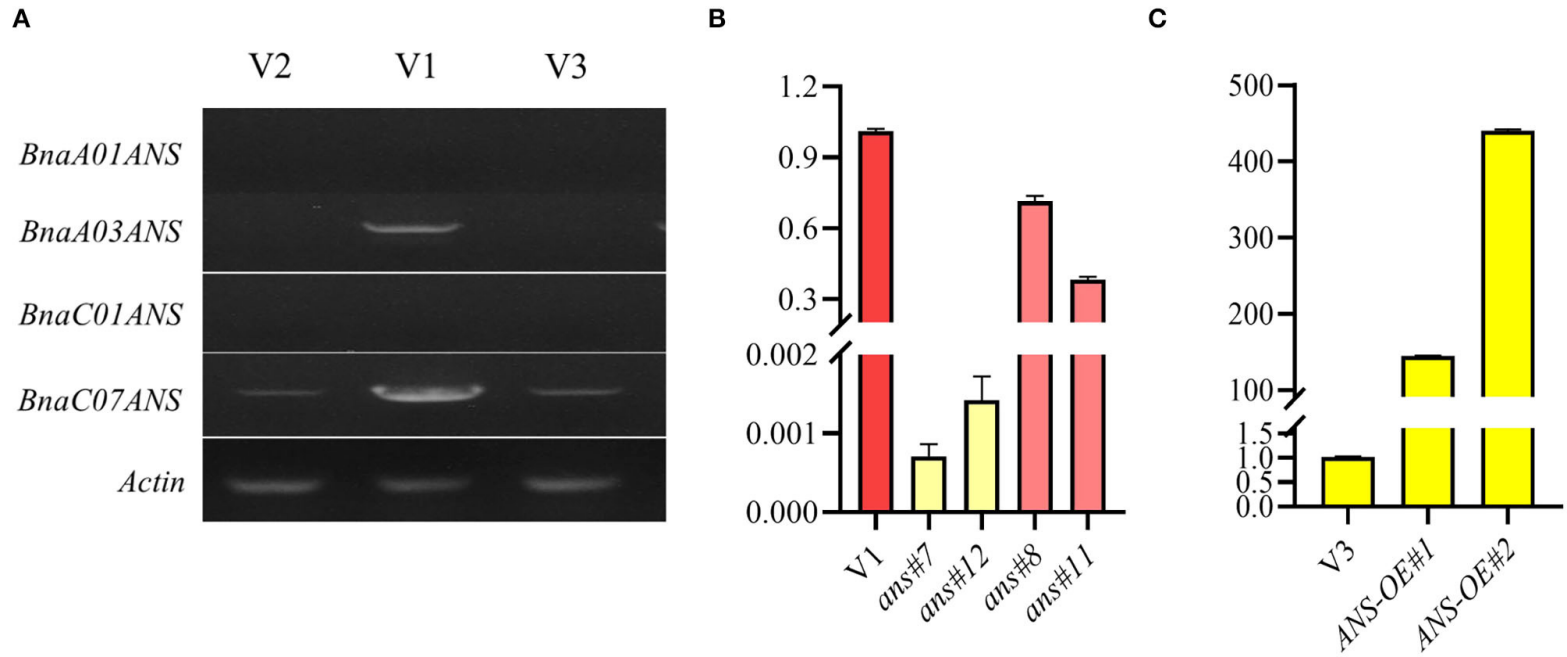
Identification of the expression level of 4 ANS copies, and the *BnaA03.ANS* expression validation in transgenic lines. **(A)** Identification of the expression level of 4 ANS copies by semi-RT-PCR; **(B)** qRT-PCR validation of *BnaA03.ANS* expression level in RNAi lines compared with the wild type (V1). **(C)** qRT-PCR validation of *BnaA03.ANS* expression level in overexpression lines compared with the wild type (V3).

Semiquantitative RT-PCR was also conducted to identify the expression level of 4 copies of *ANS* genes in V1, V2, and V3. The results showed that *BnaA03.ANS* appeared evident difference in all lines, with the brighter band in V1 (Figure 6A). Hence, *BnaA03.ANS* was chosen to be the candidate gene for RNAi and overexpression validation.

RNA interference of *BnaA03.ANS* showed that, opened petal colors of V1 turned from rose and raspberry red to zinc yellow, with the expression level of *BnaA03.ANS* decreased by 700-fold −1,400-fold, and turn to beige red when the expression level of *BnaA03.ANS* was only 71.5% or 28.25% of V3 (Figure 6B).

**Figure 7:**
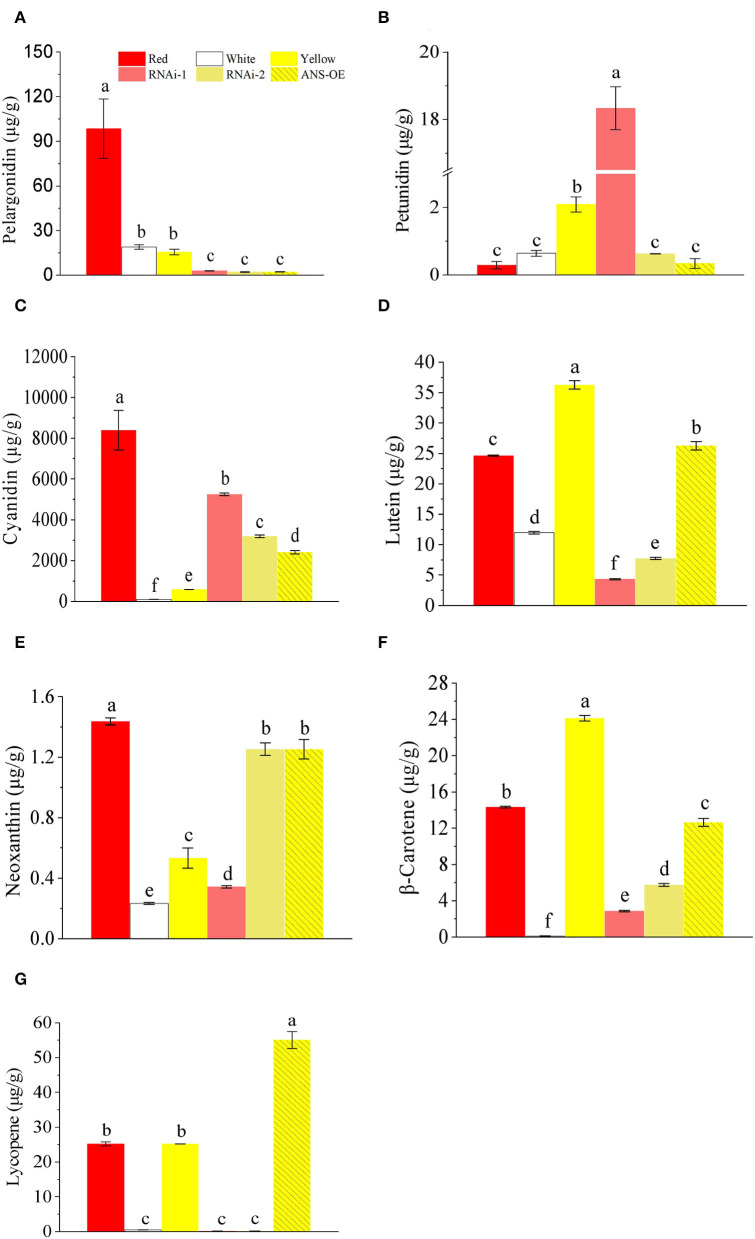
Comparison of anthocyanins and carotenoids contents in petals of red (V1), white (V2), yellow (V3), RNAi lines, and overexpression lines. **(A)** pelargonidin; **(B)** petunidin; **(C)** cyanidin; **(D)** lutein; **(E)** neoxanthin; **(F)** β-carotene; **(G)** lycopene. Letters above the columns represent a significant difference, *p* ≤ 0.05. Error bars represent SD values.

Analysis of anthocyanin and carotenoid profiles indicated that pelargonidin and cyanidin were largely decreased by 97.0% and 37.5% in *ans#7*, and 97.8% and 62.1% in *ans#8* compared with V1, respectively. As for carotenoids, lutein, neoxanthin, and β-carotene were also significantly decreased, with 82.5%, 76.2%, and 80.1% and 68.6%, 12.4%, and 60% lower in *ans#7* and *ans#8* than V1, respectively. It is interesting to note that no lycopene contents were detected when the expression of BnaA03.ANS was interferenced (Figure 7).

The full-length cDNA fragment of *BnaA03.ANS* was also overexpressed under the control of petal-specific promoter *XY355* (Fu et al., 2018), but no obvious color change appeared with *BnaA03.ANS* was overexpressed by 144-fold or 440-fold (Figure 6C). Carotenoids and anthocyanin profiles showed that cyanidin (3.15-fold), neoxanthin (1.35-fold), and lycopene (1.18-fold) were high accumulated, while, pelargonidin (-85.7%), petunidin (-83.8%), lutein (-27.6%), and β-carotene (-47.7%) were lower in *ANS-OE* than in V3. However, the petal colors did not obviously differ from yellow (Figure 7).

## Discussion

Colorful flowers are one of the most important traits of many ornamental flowering plants and have gained more and more attention from breeders (Nishihara and Nakatsuka, 2011). Flower colors in rapeseed range from white, orange to yellow, and other rare colors such as pink, purple, and red also have been reported recently, which greatly enhanced its ornamental value (Fu et al., 2018). However, the underlying mechanisms of how genes and metabolites regulate color formation in red petals have not been elucidated thoroughly (Yin et al., 2019). In this study, the DAMs and DEGs in unopened small petals (stage 1) and unopened large petals (stage 4) of red color rapeseed (V1) compared with those in white (V2) and yellow (V3) were identified, aiming at understanding the molecular mechanism on the regulation of red petal color formation in rapeseed.

### Metabolomic Analysis Revealed That Flavonoids May Contribute to Red Color Formation

The result of the principal component analysis of metabolomic research showed that two groups of red petals in rapeseed (V1-1 and V1-4) exhibited obvious separation from white and yellow ones (Figures 2A,B), which reflected that accumulation levels of metabolites in red petals have a huge difference in compared with white and yellow petals. Further analysis revealed that a total of 46+2+103 DAMs in positive mode and 20+73 DAMs in negative mode (Figure 2) were co-differentially accumulated in Co-DAMs-T1 (co-DAMs in V1 vs. V2 and V1 vs. V3 at stage 1), Co-DAMs-T4 (co-DAMs in V1 vs. V2 and V1 vs. V3 at stage 4), and group V1-1 vs. V1-4 (co-DAMs in V1 at stage 1 vs. V1 in stage 4), and these DAMs were considered as key metabolites that influence red petal formation and the differentiation from white and yellow petals. Among them, most DAMs were belonging to flavonoids (Figures 3A,B). Flavonoids, as the most important pigment, generate the widest spectrum of colors. Chen et al. (2012) reported that the flavonoid composition of white flower chrysanthemum only contained flavonols and flavones, while anthocyanins were detected in pink flowers. He et al. (2011) found that anthocyanins were presented in red and orange samples of *lycoris longituba* while no anthocyanins were detected within white and yellow. In this study, the heatmap of DAMs accumulation levels revealed that the accumulation level in red petals (V1-1 and V1-4) was higher than in white and yellow ones, with flavonoids being the most categories, which indicated the importance of flavonoids in red pigment formation compared with white and yellow.

### Identification of RNA-seq Data Showed Anthocyanin Biosynthesis Pathway and MYB, bHLH Transcription Factors Were Largely Differentiated in V1, V2, and V3

Venn diagrams of RNA-seq revealed that a total of 1,588 (163+127+1,298) genes (Figure 4) were co-differentially expressed in group Co-DEGs-T1 (co-DEGs in V1 vs. V2 and V1 vs. V3 at stage 1), group Co-DEGs-T4 (co-DEGs in V1 vs. V2 and V1 vs. V3 at stage 4), and group V1-1 vs. V1-4 (co-DEGs in V1 at stage 1 vs. V1 at stage 4), and these co-DEGs were considered as key genes that influence the red petal color formation and the differentiation from white and yellow petal colors. Anthocyanins are formed by various sugars and anthocyanidins, and the diversity of colors is highly related to anthocyanin composition and content according to a previous study (Li et al., 2003). Genes involved in the anthocyanin biosynthesis pathway that function on petal colors have been well-reported, such as *CHS* in *Malus crabapple* (Sun et al., 2015), *CHI* in asters and cyclamen (Nishihara et al., 2005), *F3H* in carnation (Owens et al., 2008), *DFR* in *Saussurea* (Li et al., 2012), and *ANS* in lisianthus flowers (Shimizu et al., 2011), which demonstrated the importance of anthocyanin biosynthesis pathway on petal color formation. As a report, red color formation, as the result of anthocyanin accumulation, is controlled through the coordination of genes that encode the enzymes involved in the anthocyanin pathway (Lai et al., 2020). For example, in radish of Cruciferae, genes for the anthocyanin biosynthesis, namely, *PAL, C4H, 4CL, CHS, CHI, F3H, DFR*, and *ANS*, were identified by traditional manners and second-generation sequencing (Muleke et al., 2017; Sun et al., 2018). Among them, *RsF3H, RsF3*'*H1, RsCHS3, RsANS*, and particularly *RsUFGT*, were highly correlated with the anthocyanin contents in the flesh of red radish (Muleke et al., 2017). However, when both the skin and flesh were factored into account, only the expression of *RsDFR* and *RsANS* were correlated with the anthocyanin contents (Park et al., 2011). These results indicated that late structural genes involved in the flavonoid pathway are specifically involved in anthocyanin biosynthesis. According to our research, flavonoids were also highly accumulated from metabolomics data, and the genes and pathway involved in anthocyanin biosynthesis were drawn against these Co-DEGs-red (163+127+1,298). According to Figure 5, genes showed a diverse difference between red with white and yellow, while *ANS, DFR*, and *UF3GT* were greatly highly expressed in red petals than in white and yellow, and these results were further validated by qRT-PCR (Supplementary Figure 2).

In addition to these structural genes, transcription factors also play a role in petal color formation. MYB, bHLH, and WD40 families are reported to be the three major types of transcription factors that are involved in petal coloration and also regulate anthocyanin synthesis directly or indirectly (Zhang et al., 2003; Ramsay and Glover, 2005), for instance, the bleaching of gentian due to mutation of *GtMYB3* (Nakatsuka et al., 2008), and also the positive regulation function of *GhMYB10* and *LhMYB6* on anthocyanin accumulation in gerbera and lily (Roosa et al., 2008; Yamagishi et al., 2010). Overexpression of an R2R3-MYB transcription factor, *RsMYB1*, resulted in the increasing production of red flowers in radish, and by ectopic overexpression of the *OvPAP2* (*Orychophragmus violaceus*) gene, Fu et al. (2018) successfully produced red anthers and petals in yellow rapeseed (Lim et al., 2016; Fu et al., 2018). As for bHLH, in *Arabidopsis*, three bHLH transcription factors, GL3, TT8, and EGL3 were reported to participate in the biosynthesis of flavonoids (Nesi et al., 2000; Baudry et al., 2006). A recent report showed that the coexpression of RSMYB1 and RsTT8 in tobacco leaves remarkably increased the accumulation of anthocyanins, indicating the partnership of RsTT8 and RsMYB1 in anthocyanin biosynthesis (Lim et al., 2017). To unveil the mechanisms of red color formation, the two-most important regulators involved in anthocyanin biosynthesis were isolated, and the expression module of MYB and bHLH were drawn in Supplementary Figure 2, which showed huge differences in red petals than in white and yellow at stage 1 or stage 2, reflecting the importance of MYB and bHLH on red color formation in rapeseed.

### RNA Interference of *BnaA03.ANS* Transferred Petal Color From Raspberry Red to Beige Red or Zinc Yellow, While no Obvious Petal Color Was Changed When *BnaA03.ANS* Was Overexpressed

According to metabolomics and RNA-seq data, aiming at flavonoids, we selected the 4 copies of *ANS* genes that played a key role in the anthocyanin biosynthesis pathway, and semi-RT-PCR was performed. The results showed that band of *BnaA03.ANS* in red petals exhibited an obvious difference from yellow and white petals compared with the other 3 copies, which indicates the importance of *BnaA03.ANS*. Disruption of *BnaA03.ANS* in red rapeseed led to converting the petal color from raspberry red to beige red when 28.5% or 71.7% of the expression level of *BnaA03.ANS* was suppressed, while the color changed to zinc yellow, when 99.93% or 99.86% of the expression level was suppressed. The result of anthocyanins and carotenoids contents analysis revealed that pelargonidin, cyanidin, lutein, neoxanthin, β-carotene, and lycopene contents were significantly inhibited (Figure 7), which indicated the importance of *BnaA03.ANS* on red petal formation. However, with the overexpression of *BnaA03.ANS* under XY355 promoter in yellow rapeseed, no change of petal color was detected, though the pigments showed a different change with cyanidin, neoxanthin, and lycopene content highly accumulated while pelargonidin, petunidin, lutein, and β-carotene were decreased in overexpression lines than control (yellow rapeseed). According to a previous report, even flowers of the same color exhibited distinct pigment profiles depending on the species and variety, for example, in yellow tomato flowers, neoxanthin and violaxanthin comprise the two major carotenoids, whereas 9-cis-violaxanthin is the predominant component in yellow rose flowers (Ariizumi et al., 2014; Wan et al., 2019; Liu et al., 2020). Therefore, further researches still need to be investigated.

## Data Availability Statement

The datasets presented in this study can be found in online repositories. The names of the repository/repositories and accession number(s) can be found at: https://www.ncbi.nlm.nih.gov/bioproject/PRJNA848086.

## Author Contributions

SH, HL, and LJ designed the experiment. PH, HL, BL, YR, and LH performed the experiment. PH and SH wrote the manuscript. All authors read and agreed to the final manuscript.

## Funding

This work was supported by the earmarked fund for the China Agriculture Research System (CARS-12), the Zhejiang Natural Science Foundation (Y21C13014), the National Natural Science Foundation (32130076), the Zhejiang Science and Technology Major Program on Agricultural New Variety Breeding (2021C02064), and the Zhejiang Key Laboratory of Digital Dry Land Crops (2022E10012).

## Conflict of Interest

The authors declare that the research was conducted in the absence of any commercial or financial relationships that could be construed as a potential conflict of interest.

## Publisher's Note

All claims expressed in this article are solely those of the authors and do not necessarily represent those of their affiliated organizations, or those of the publisher, the editors and the reviewers. Any product that may be evaluated in this article, or claim that may be made by its manufacturer, is not guaranteed or endorsed by the publisher.

## Supplementary Material

Supplementary Figure 1 KEGG analysis of co-DEGs relative to red pigment formation.

Supplementary Figure 2 Heatmap of bHLH and MYB transcription factors among co-DEGs relative to red pigment formation.

Supplementary Figure 3 qRT-PCR identification of genes relative to anthocyanin biosynthesis.

Supplementary Table 1 Primers used in this research.

Supplementary Table 2 Basic results of RNA-seq data.
