# Supplementary material for: BnaA03.ANS Identified by Metabolomics and RNA-seq Partly Played Irreplaceable Role in Pigmentation of Red Rapeseed (Brassica napus) Petal
Source: Front Plant Sci. 2022 Jul 14;13:940765. doi: 10.3389/fpls.2022.940765 (PMC9330612; doi:10.3389/fpls.2022.940765)
Supplement: Supplementary Figure 1 — KEGG analysis of co-DEGs relative to red pigment formation. [file Presentation_1.PPTX]

## Slide 1
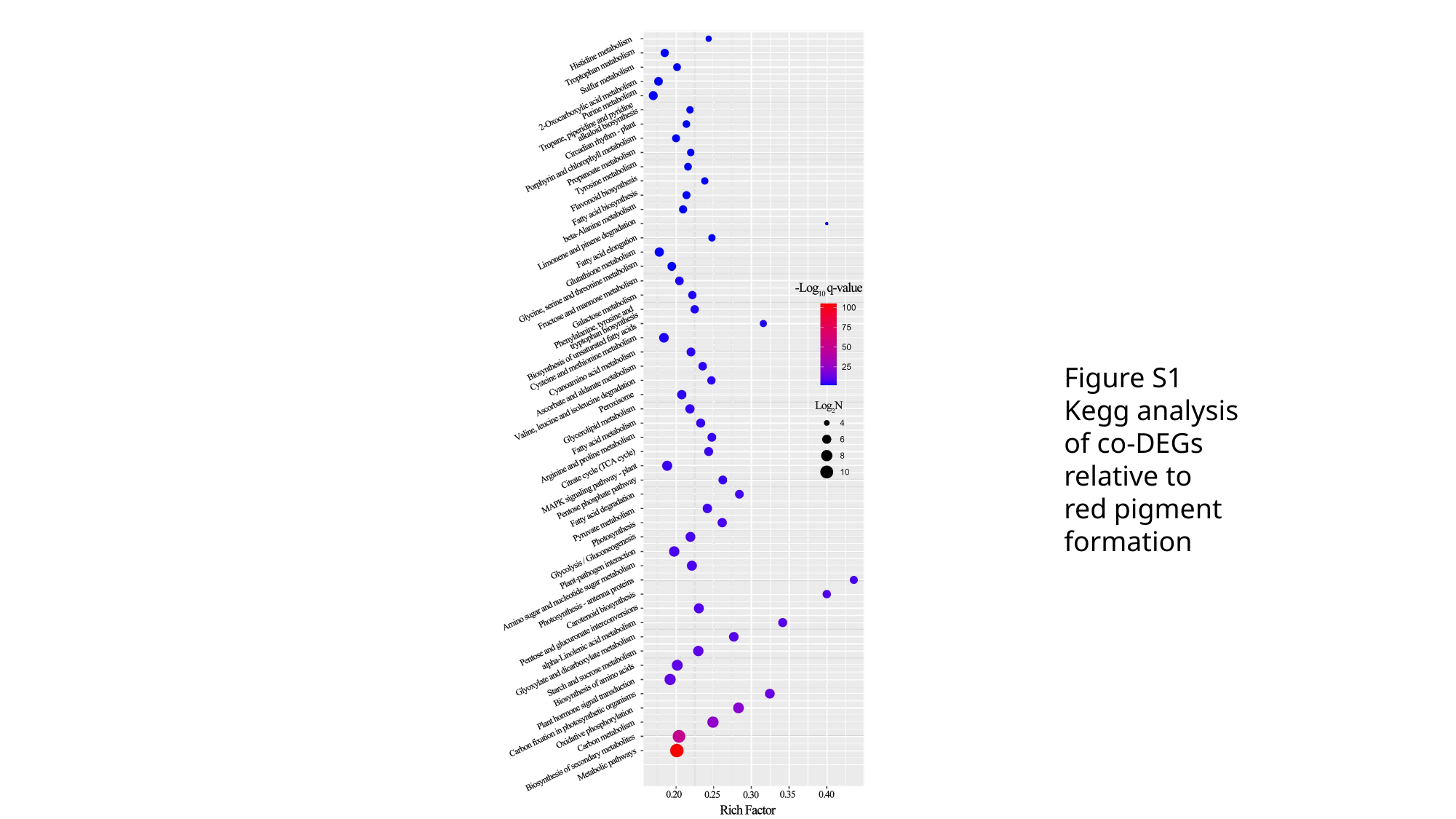

Figure S1 Kegg analysis of co-DEGs relative to red pigment formation

## Slide 2
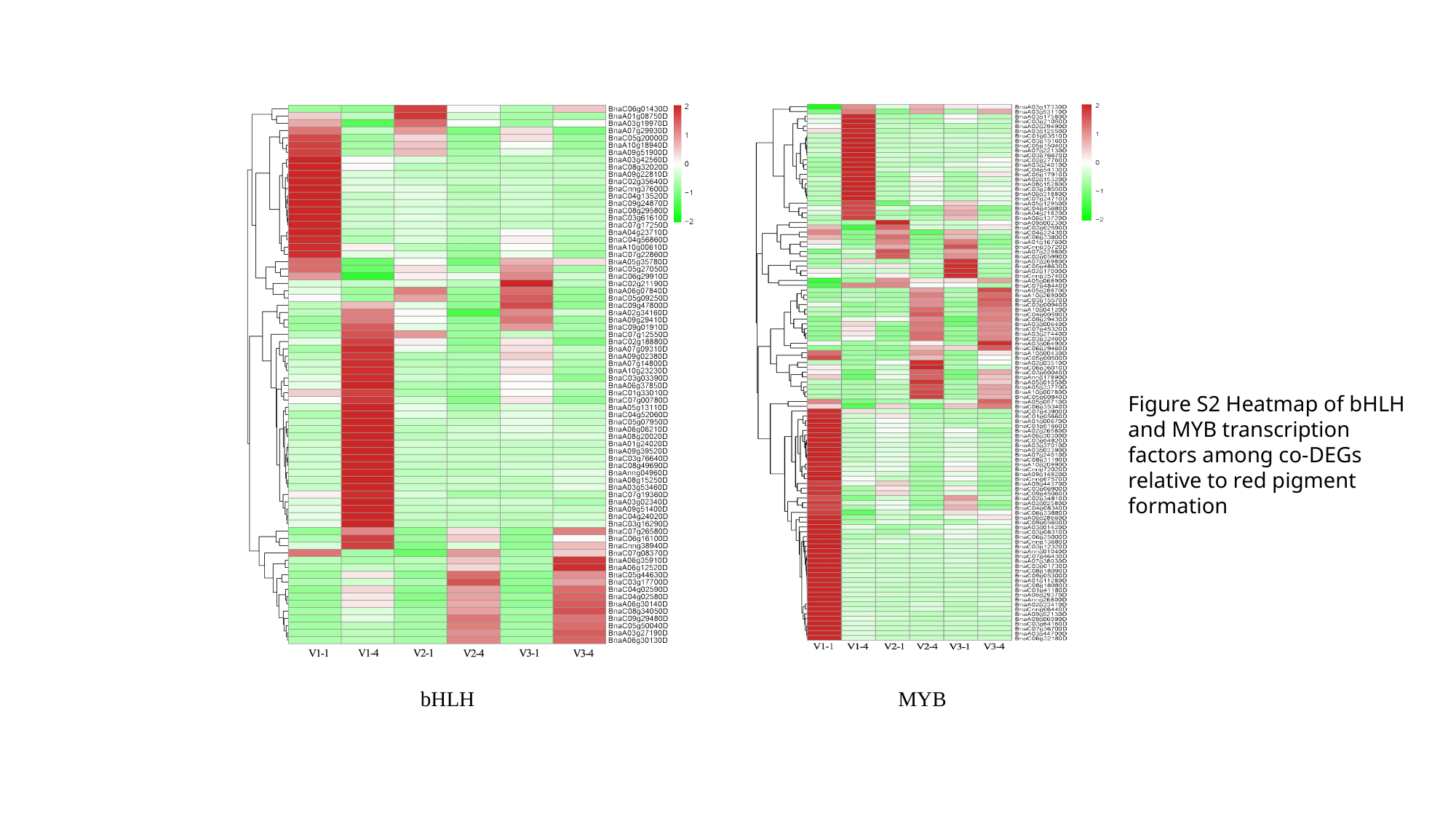

Figure S2 Heatmap of bHLH and MYB transcription factors among co-DEGs relative to red pigment formation
bHLH
MYB

## Slide 3
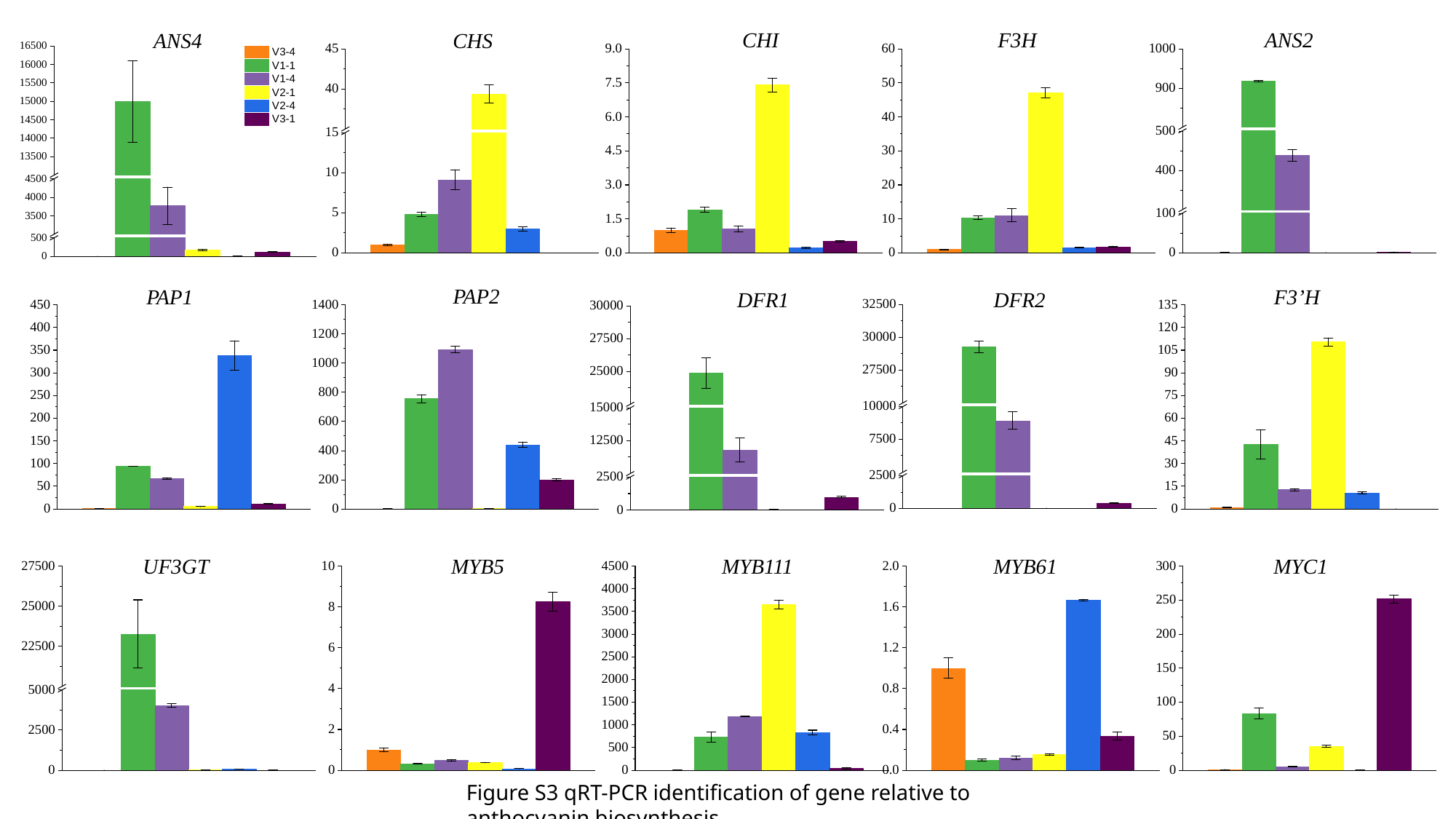

CHI
F3H
ANS2
CHS
ANS4
PAP2
PAP1
F3’H
DFR1
DFR2
MYB5
MYB111
MYB61
MYC1
UF3GT
Figure S3 qRT-PCR identification of gene relative to anthocyanin biosynthesis.
